# Das Ansehen von Hausfrauen in Deutschland – Eine quantitativ-empirische Analyse differenzieller Wahrnehmungen

**DOI:** 10.1007/s11577-022-00819-6

**Published:** 2022-04-12

**Authors:** Katrin Stache, Christian Ebner, Daniela Rohrbach-Schmidt

**Affiliations:** 1 Institut für Soziologie, Lehrstuhl für Soziologie mit Schwerpunkt Arbeit und Organisation, Technische Universität Braunschweig, Bienroder Weg 97, 38106 Braunschweig, Deutschland; 2 Forschungsdatenzentrum (BIBB-FDZ), Bundesinstitut für Berufsbildung, Robert-Schuman-Platz 3, 53175 Bonn, Deutschland

**Keywords:** Prestige, Nichterwerbstätigkeit, Soziale Ungleichheit, Reproduktionsarbeit, Hausarbeit, Reputation, Nonemployment, Social inequality, Recognition, Housework

## Abstract

Die Erwerbstätigenquote von Frauen ist in den letzten Jahrzehnten international wie auch in Deutschland merklich angestiegen, wohingegen das klassische *Male Breadwinner Model* zunehmend erodiert. Vor diesem Hintergrund hat die Studie zum Ziel, das gesellschaftliche Ansehen der zahlenmäßig geringer werdenden Gruppe von Hausfrauen, deren Haupttätigkeit in der Haus- und Familienarbeit liegt, mit aktuellen Befragungsdaten zu untersuchen. Die Analysen gehen zum einen der Frage nach, wie hoch das Ansehen generell von der Bevölkerung in Deutschland eingestuft wird und zum anderen, ob sich die Wahrnehmungen anhand von ausgewählten soziodemografischen Merkmalen systematisch unterscheiden. Die empirischen Befunde zeigen, dass das Ansehen von Hausfrauen in Deutschland im Allgemeinen höher eingeschätzt wird als das von Arbeitslosen sowie von Helfertätigkeiten, aber auch geringer als das Ansehen von beruflichen Tätigkeiten auf Fachkräfteniveau. Darüber hinaus variieren die Beurteilungen zum Hausfrauenprestige signifikant danach, welcher sozialen Gruppe (Geburtskohorte, Ausbildungsniveau, Erwerbsbeteiligung, Geschlecht) die Befragten angehören. Weiterführende Analysen von Interaktionseffekten verdeutlichen ferner ein differenziertes Zusammenspiel der Variable Geschlecht mit anderen Gruppenmerkmalen. Der Beitrag schließt mit einer ausführlichen Diskussion der Ergebnisse sowie einem Ausblick auf zukünftige Forschung.

## Abstract (en)

The employment rate of women has increased markedly over the past decades, both internationally and in Germany, whereas the classic male breadwinner model is eroding. Against this background, using current survey data, the aim of this study is to examine the social prestige of the shrinking group of housewives whose main activity is domestic and family work. The analyses address, on the one hand, the question of how high their reputation is generally rated by the population in Germany and, on the other hand, whether the perceptions differ systematically by raters’ sociodemographic characteristics. The empirical findings show that the reputation of housewives in Germany is generally rated higher than that of the unemployed and of those in helper jobs but lower than for those in professional jobs at the skilled level. Moreover, the assessments of housewives’ prestige vary significantly according to the social group (birth cohort, level of education, labor force participation, gender) to which the respondents belong. Further analyses of interaction effects also reveal a differentiated interaction of the gender variable with the other structural group characteristics. The paper concludes with a detailed discussion of the results and an outlook on future research.

## Einleitung

In den letzten Jahrzehnten ist die Frauenerwerbstätigenquote in großen Teilen Europas deutlich angestiegen (ILO 2016, S. 6, 8 f.).^1^ In Deutschland ist dieser Trend ebenfalls klar erkennbar: Die Zahl erwerbstätiger Frauen hat seit 1991 um mehr als 20 % zugenommen (Wanger 2017, S. 27 f.), und auch Mütter sind immer häufiger auf dem Arbeitsmarkt vertreten (BMFSFJ 2012, S. 26 ff., 2017, S. 66 f.). Das traditionelle männliche Alleinernährermodell, welches in der Vergangenheit noch stärker institutionell begünstigt wurde, erodiert somit zunehmend (vgl. Gärtner et al. 2020). Diese Entwicklung führt unweigerlich zu Veränderungen im familiären Zusammenleben und oft zu Spannungen zwischen Erwerbs‑, Erziehungs- und Hausarbeit. Frauen tragen noch immer die Hauptlast der Reproduktionsarbeit und sind somit stärker von einem Vereinbarkeitsdilemma betroffen (vgl. Gärtner et al. 2020; Geißler 2014, S. 398 ff.; Haberkern 2007; Becker-Schmidt 2008). Nur etwa 10 % der Mütter haben zudem selbst den Eindruck, dass ihr Engagement von der Gesellschaft anerkannt wird (IfD Allensbach 2013, S. 75, 79). Gesellschaftliche und politische Diskussionen drehen sich daher in jüngster Zeit vor allem um Fragen der institutionellen Gleichstellung von Männern und Frauen sowie der Vereinbarkeit von Beruf und Familie. Damit einhergehend erfährt die soziale Position der Hausfrau mutmaßlich einen Ansehensverlust (Meyer 2014, S. 439). So lehnt die Mehrheit der Familien heute eine dauerhafte Spezialisierung von Haus- und Familienarbeit auf der einen und Erwerbsarbeit auf der anderen Seite ab und bevorzugt stattdessen eine partnerschaftliche Arbeitsteilung (vgl. IfD Allensbach 2015; Ebner et al. 2020). Das Frauenbild verändert sich insgesamt durch Forderungen nach Geschlechtergleichberechtigung, einem Wandel kultureller Werteordnungen (vgl. Inglehart und Norris 2003), Individualisierungstendenzen (vgl. Beck und Beck-Gernsheim 1990; Beck-Gernsheim 1983) sowie einer Pluralisierung von Lebens- und Beziehungsformen (vgl. Nave-Herz 2015; Meyer und Schulze 1993).

Während sich eine Vielzahl der einschlägigen Forschungsliteratur mit der Arbeitsteilung zwischen Männern und Frauen oder Einstellungen gegenüber geschlechtsspezifischen Rollenverteilungen auseinandersetzt, steht eine Erfassung des gesellschaftlichen Ansehens von Hausfrauen auf Basis quantitativer Befragungsdaten für Deutschland bis heute jedoch aus. In der internationalen Prestigeforschung wurde zwar schon früh auf die Relevanz eines eigenen Hausfrauenstatus hingewiesen (vgl. Dworkin 1981). Dennoch existieren bis dato kaum Arbeiten hierzu (siehe aber für die USA: Nilson 1978; Bose 1985), da der empirische Fokus hauptsächlich auf der Untersuchung des Sozialprestiges unterschiedlicher Berufe lag (u. a. Treiman 1977; Ganzeboom und Treiman 1996; Wegener 1985).

Das Ansehen einer Person gibt einen entscheidenden Hinweis auf ihre hierarchische Stellung innerhalb der Sozialstruktur, bestimmt also mit über ihren gesellschaftlichen Status. Hoch angesehen zu sein, kann Vorteile in alltäglichen Interaktionen und Austauschsituationen mit sich bringen und zu gesellschaftlichem Einfluss verhelfen (Homans 1968). Insofern stellt Ansehen oder Prestige als symbolisches Kapital (Bourdieu 1983) eine wertvolle individuelle Ressource dar. Einerseits müssen sich Individuen ihr Ansehen verdienen; andererseits wird es durch gesellschaftliche Attributionsprozesse mit sozialen Positionen in Verbindung gebracht. Unterschiedliche Berufsgruppen beispielsweise können in ihrem Prestigewert stark variieren (vgl. Treiman 1977; Wegener 1985). Für Positionen, deren Vertreter die nichterwerbstätige Bevölkerung repräsentieren (z. B. Hausfrauen oder Arbeitslose), dürften Prestigezuschreibungen zu ähnlichen Vor- oder Nachteilen im gesellschaftlichen Zusammenleben führen.

Ziel dieser Studie ist es, Aufschluss darüber zu geben, wie die deutsche Bevölkerung das gesellschaftliche Ansehen von Hausfrauen beurteilt. Zunächst sollen Hausfrauen relativ zu anderen sozialen Positionen in der Statushierarchie des Landes verortet werden. Darüber hinaus soll geprüft werden, ob die Statuswahrnehmungen der befragten Personen je nach Zugehörigkeit zu sozialen Gruppen variieren. Damit sind die folgenden beiden forschungsleitenden Fragen für die vorliegende Untersuchung maßgeblich:

1. Wie angesehen ist die Hausfrauentätigkeit im Vergleich zu Arbeitslosigkeit und Erwerbstätigkeit?
  Vor dem Hintergrund der gestiegenen Frauenerwerbstätigenquote sind Hausfrauen mehr und mehr zu einer marginalisierten Gruppe geworden. Es werden aber auch negative Assoziationen mit der weiblichen Karriere verbunden (vgl. Diabaté 2015) und Retraditionalisierungstrends wahrgenommen (Geißler 2014, S. 408), welche sich in einer, zumindest zeitweise, gewollt traditionellen Ausrichtung der Arbeitsteilung in der Familie ausdrücken. Diese scheinbar gegensätzlichen Anhaltspunkte machen eine Bestandsaufnahme des Hausfrauenprestiges besonders interessant. Dabei geht es auch um die vertikale Verortung der Hausfrauentätigkeit neben Arbeitslosigkeit auf der einen Seite und Erwerbstätigkeit auf der anderen Seite.
2. Gibt es Unterschiede in den Einschätzungen zum Ansehen von Hausfrauen in Abhängigkeit sozialstruktureller Merkmale der Beurteilenden?
  Klassische Studien aus dem Bereich der Berufsprestigeforschung bekräftigten immer wieder die große Übereinstimmung in den Urteilen der Befragten bei der hierarchischen Verortung von Berufen (z. B. Hodge et al. 1964; Nakao und Treas 1994; Treiman 1977). Diese Perspektive blieb jedoch nicht unumstritten (z. B. Boltanski und Thévenot 1983). So konnten etwa systematisch unterschiedliche Wahrnehmungen beruflichen Prestiges entlang von Personenmerkmalen wie Bildung (z. B. MacKinnon und Langford 1994; Wegener 1992) oder Geschlecht (z. B. Valentino 2020) empirisch klar nachgewiesen werden. Und auch theoretisch ist es gut zu begründen, dass die Wahrnehmungen hinsichtlich des Hausfrauenprestiges nicht universeller Natur sind, sondern von bestimmten Personenmerkmalen der Befragten abhängen. Folglich werden die Einschätzungen nach Geschlecht und Ausbildungsstand sowie nach Geburtsjahrgang und Teilhabe am Erwerbssystem differenziert untersucht. Es ist ferner anzunehmen, dass Männer und Frauen die Position der Hausfrau unterschiedlich wahrnehmen, weil sie jeweils durch geschlechterspezifische Leitbilder und Rollenerwartungen geprägt sind. Die Geschlechtervariable wird aus diesem Grund nicht (nur) isoliert, sondern im Zusammenspiel mit den beschriebenen Strukturmerkmalen (Geburtsjahrgang/‑kohorte, Ausbildungsniveau, Erwerbsbeteiligung) detaillierter untersucht.

Mit der Beantwortung dieser Ausgangsfragen tragen wir in mehrfacher Hinsicht zur Erweiterung des Forschungsstands bei. Erstens schaffen wir neue Erkenntnisse jenseits von Studien zur Erklärung geschlechtsspezifischer Ungleichheit,^2^ die sich mit der tatsächlichen Aufteilung von Reproduktions- und Erwerbsarbeit zwischen den Geschlechtern, dahinterliegenden Gründen oder Einstellungen gegenüber partnerschaftlicher Arbeitsteilung befassen, indem wir explizit die Prestigedimension in den Blick nehmen. Zweitens ergänzen wir den Forschungsstand zum Ansehen von Berufsgruppen sinnvoll; mit dem Fokus auf Hausfrauen analysieren wir nun auch das Ansehen einer Gruppe der nichterwerbstätigen Bevölkerung auf Basis einer aktuellen quantitativen Erhebung für Deutschland. Wir liefern erste Hinweise darauf, wie sich das Ansehen von Hausfrauen im Vergleich zu anderen sozialen Positionen darstellt, insbesondere zu Arbeitslosen als einer weiteren Gruppe Nichterwerbstätiger sowie zu beruflichen Tätigkeiten mit unterschiedlichen Anforderungsniveaus. Und drittens erweitern wir das Verständnis gesellschaftlicher Konstruktion von Status; hier gemessen anhand von Einschätzungen aus der Bevölkerung zum Prestige der sozialen Position Hausfrau. Anders als in der Berufsprestigeforschung häufig postuliert (vgl. Nakao 2020), gehen wir nicht davon aus, dass eine konsensuelle, allgemeinhin geteilte Meinung hierzu existiert. Durch die differenzierte Analyse der aktuellen Datenbasis können gegebenenfalls gruppenspezifische Wahrnehmungen zum Status der Hausfrau sowie Interaktionseffekte aufgedeckt werden, die sich an der sozialen Realität der jeweiligen Gruppenmitglieder orientieren.

Im folgenden Abschnitt (Abschn. 2) skizzieren wir mit den Ursachen für die gestiegene Frauenerwerbstätigkeit in Deutschland sowie einem Blick auf die gesellschaftliche Anerkennung von Reproduktionsarbeit zunächst die Ausgangslage der Thematik und formulieren auf Grundlage unterschiedlicher theoretischer Perspektiven sowie empirischer Vorkenntnisse Forschungshypothesen für unsere Analysen. Im Anschluss (Abschn. 3) erläutern wir das Forschungsdesign der Studie. Auf die Beschreibung der Datengrundlage und des Samples folgen die Vorstellung der zentralen Variablen der Untersuchung sowie die Beschreibung der Analysestrategie. Im Ergebnisteil (Abschn. 4) präsentieren wir zentrale Befunde, sowohl zum Ansehen von Hausfrauen im Allgemeinen als auch differenziert nach sozialstruktureller Gruppenzugehörigkeit. Der Beitrag schließt mit einem Fazit und dem Ausblick auf zukünftige Forschungsfragen, welche sich an die vorliegende Studie anschließen (Abschn. 5).

## Hausfrauen in Deutschland und deren Ansehen – Theoretischer Hintergrund, Forschungsstand und Hypothesen

### Die Entwicklung der Frauenerwerbstätigkeit in Deutschland

1961 betrug die weibliche Erwerbstätigenquote in der Bundesrepublik rund 33 %, die männliche rund 64 % (Statistisches Bundesamt 1967, S. 28). Im Jahr 1991 waren bereits rund 57 % der Frauen im erwerbsfähigen Alter auf dem Arbeitsmarkt im wiedervereinigten Deutschland vertreten, 2019 lag ihr Anteil sogar bei rund 73 %, derjenige der Männer bei rund 81 % (Destatis 2019a). Diese Entwicklung kann als Wechselspiel aus institutionellen Reformen und kulturellem Wandel aufgefasst werden (vgl. Beck-Gernsheim 1983), was ohne die seit Mitte des 19. Jahrhunderts aufkommende Frauenbewegung in den westlichen Industrienationen wohl kaum denkbar gewesen wäre (vgl. Sommerkorn 1988). Im Speziellen lassen sich für die Zunahme der Erwerbsbeteiligung von Frauen folgende Einflussfaktoren identifizieren:

Auf der Ebene individueller Qualifikationen kann über die Jahrzehnte hinweg eine stetige Verbesserung der Bildungsergebnisse von Frauen und eine Angleichung der beruflichen Abschlüsse zwischen den Geschlechtern beobachtet werden (vgl. Geißler 2014, S. 375 ff.; Beck-Gernsheim 1983, S. 19 ff.). Damit sind auch die Arbeitsmarktchancen der weiblichen Bevölkerung weiter angestiegen. Veränderte Einstellungen und Wertorientierungen betonen Erwerbsarbeit als persönliches Anliegen und Möglichkeit der Selbstentfaltung für das weibliche Geschlecht (vgl. Beck und Beck-Gernsheim 1990; Beck-Gernsheim 1983). Aber auch Männer sehen im Einklang mit einem allgemeinen Anstieg postmaterialistischer Wertorientierungen eine Beteiligung von Frauen am Arbeitsmarkt heute seltener als problematisch an (vgl. Inglehart 1998). Auf der Haushaltsebene treten immer häufiger ökonomische Zwänge auf, die Doppelverdienerhaushalte begünstigen (vgl. Sommerkorn 1988; Dressel und Wanger 2010, S. 489). So reicht das Einkommen des Mannes, beispielsweise durch die Ausweitung des Niedriglohnsektors seit Ende der 1990er-Jahre, in vielen Fällen nicht mehr aus, um die Familie zu ernähren (vgl. Kalina und Weinkopf 2020, S. 7). Mit einer (Teilzeit‑)Erwerbstätigkeit der Frau kann ein zusätzlicher Beitrag zum Familieneinkommen geleistet werden. Aus demografischer Perspektive hat der Rückgang der Geburten nach dem Babyboom der 1960er-Jahre (vgl. Meyer und Schulze 1993, S. 170 f.; Destatis 2021a, 2019b) und damit zusammenhängend auch ein geringeres Maß an Erziehungsarbeit womöglich Einfluss auf die Chancen von Frauen, einer außerhäuslichen Berufstätigkeit nachzugehen (vgl. Bianchi et al. 2000). Zudem sind Eheschließungen seit den 1950er-Jahren stark rückläufig (Destatis 2021b) und Scheidungen nehmen tendenziell zu (vgl. Meyer und Schulze 1993, S. 169 ff.; Wagner et al. 2015; BMFSFJ 2017, S. 41), was der Notwendigkeit einer Erwerbstätigkeit von Frauen Vorschub leistet, weil diese sich dadurch nicht mehr auf das Einkommen ihres Partners verlassen können. Der Wandel der Branchen- und Berufsstruktur begünstigt weibliche Erwerbsbeteiligung insbesondere durch die wachsende Bedeutung von Dienstleistungen (Tertiärisierung). Traditionell liegt der Frauenanteil im Dienstleistungssektor weit über dem der Männer, unter anderem weil sich Branchen wie das Gesundheits‑, Sozial- und Erziehungswesen mit typischen Berufswünschen von Frauen decken (vgl. BA 2019a, S. 12; Dölling 1993, S. 33). Technologische Entwicklungen (Waschmaschine, Geschirrspüler etc.) haben zu einer Entlastung bei der Hausarbeit geführt (vgl. Heisig 2011), die es Frauen ermöglicht, mehr Zeit in entlohnte Tätigkeiten zu investieren. Auf rechtlicher Ebene wurden gesetzliche Einschränkungen der Frauenerwerbsarbeit im BGB allmählich aufgehoben. Vor der Reform des Ehe- und Familienrechts im Jahr 1976 bestand in der BRD das Leitmodell der Hausfrauenehe: Verheiratete Frauen durften nur dann einer Erwerbsarbeit nachgehen, wenn dies mit ihren Pflichten in Ehe und Familie vereinbar war (vgl. Geißler 2014, S. 398; Dressel und Wanger 2010, S. 489). Durch die Aufhebung solcher legislativen Barrieren erlangten die Frauen zunehmend Autonomie über Entscheidungen zur Erwerbstätigkeit und gewannen darüber hinaus ökonomische Unabhängigkeit von ihrem Ehepartner.^3^ Auf infrastruktureller Ebene ist vor allem der Ausbau von Kinderbetreuungseinrichtungen zu nennen, wodurch Problemen in der Vereinbarkeit von Beruf und Familie in Teilen vorgebeugt werden kann. Je besser die infrastrukturellen Gegebenheiten sind, desto eher können sich beide Partner auf die berufliche Karriere konzentrieren. Schließlich tragen die zunehmende Flexibilisierung und Entgrenzung von Arbeit (Voß 1998) dazu bei, individuelle Lebensplanungen mit Erwerbsarbeit in Einklang bringen zu können, etwa durch Möglichkeiten der flexiblen Anpassung von Arbeitszeiten und -orten. Frauen nehmen vor allem Teilzeitarrangements deutlich häufiger in Anspruch als Männer (vgl. BA 2019a, S. 14).

Mit dem Anstieg der Frauenerwerbsbeteiligung nimmt reziprok die Zahl der Hausfrauen im langfristigen Trend ab. Eine zahlenmäßige Erfassung dieser Gruppe ist in offiziellen Statistiken allerdings nicht direkt vorgesehen, was auch damit zusammenhängt, dass hierfür keine eindeutige Definition existiert. Sehr häufig geht die Hausfrauentätigkeit vermutlich mit einer Mutterschaft einher. Demnach könnten vor allem rund 2,6 Millionen nichterwerbstätige Mütter (218.000 Erwerbslose und 2,4 Mio. Nichterwerbspersonen im Alter von 15 bis 64 Jahren; vgl. Destatis 2019b, S. 117; Stand 2018) als Hausfrau betrachtet werden. In dieser Studie werden all diejenigen Frauen als Hausfrauen bezeichnet, die sich hauptsächlich mit Haus- und Familienarbeit, sogenannter Reproduktionsarbeit, befassen und darüber hinaus keiner (oder nur geringfügiger) Lohnarbeit nachgehen (vgl. Abschn. 3.2).

### Stellenwert der Reproduktionsarbeit und das Ansehen von Hausfrauen

Die Arbeit im Haushalt, die Erziehung und Betreuung von Kindern sowie die Pflege von Angehörigen (Sorgearbeit) sind für den Fortbestand jeglicher Gesellschaft fundamental und waren in Deutschland traditionell vor allem die Angelegenheit von (Haus‑)Frauen (vgl. Meier-Gräwe 2015; Becker-Schmidt 2008). Obgleich dieser Tatsache wendete sich die quantitative Ungleichheitsforschung insbesondere den Erwerbstätigen zu und die Gruppe der Hausfrauen blieb lange außen vor (vgl. Dworkin 1981). So wurden Berufsklassifikationen entwickelt, die zwar in höchstem Detail berufliche Tätigkeiten differenzieren, aber für die Arbeit außerhalb des Erwerbssystems keine Kategorie bereitstellen. In Deutschland etablierten sich die Klassifikationen der Berufe von 1988, 1992 und aktuell 2010. Statusmaße wie die EGP-Klassen (Erikson et al. 1979), der International Socio-Economic Index of Occupational Status (ISEI; Ganzeboom et al. 1992) oder auch Erhebungen zu sozialem Prestige (z. B. Wegener 1985) basieren jedoch in der Regel auf solchen Berufsklassifikationen (Christoph et al. 2020). Konkret bedeutete dies, dass vor allem (erwerbstätigen) Männern von wissenschaftlicher Seite überhaupt ein Ansehen zugeschrieben wurde, welches schließlich auch den sozialen Status der Familie bestimmte (vgl. Nilson 1978; Tyree und Hicks 1988). Die gesellschaftliche Stellung der Frau wurde „allein von ihrer familialen und nicht von ihrer beruflichen Rolle her definiert und geprägt“ (Sommerkorn 1988, S. 118). Arbeiten, die sich in der Vergangenheit dennoch mit der Verortung von Hausfrauen im Statusgefüge beschäftigt haben, weisen für diese Gruppe einen mittleren Prestigewert bei hoher Varianz aus (Dworkin 1981; Nilson 1978; Bose 1985), was wiederum im Zusammenhang mit Unsicherheiten bei ihrer Einordnung erklärt wurde (vgl. Tyree und Hicks 1988). Für Deutschland gibt es zum Ansehen von Hausfrauen bis dato keine Zahlen.

Dass der Ausübung einer vergüteten beruflichen Tätigkeit in Deutschland gegenüber unbezahlter Haus- und Familienarbeit ein vergleichsweise hoher Stellenwert zuerkannt wird, zeigt sich auch außerhalb der Sozialforschung in mehrfacher Hinsicht. So sind etwa (wohlfahrts-)staatliche Institutionen eng an Erwerbsarbeit gekoppelt, im Speziellen an das klassische Normalarbeitsverhältnis (vgl. Berger und Moldaschl 2006, S. 657; Mückenberger 1985). Die Hausfrauentätigkeit entspricht zudem nicht der verbreiteten Norm einer erfolgsorientierten Leistungsgesellschaft (vgl. Neckel 2001) und für Reproduktionsarbeit bedarf es im Vergleich zu einem großen Teil der entlohnten Beschäftigungen keiner formalen Qualifikation.

Während also anzunehmen ist, dass Hausfrauen im Vergleich zu beruflichen Tätigkeiten ein im Durchschnitt geringeres Ansehen zugeschrieben wird, fällt der Vergleich mit einer anderen Nichterwerbstätigengruppe, den Arbeitslosen, womöglich anders aus. Ihnen haftet das Stigma an, keiner gesellschaftlich anerkannten Arbeit nachzugehen (vgl. Goffman 1975; Gurr und Lang 2018; Hirseland und Ramos Lobato 2014). Für arbeitslose Personen ist es daher besonders schwer, aus ihrer Position heraus überhaupt gesellschaftliches Ansehen zu erlangen.

Auf Grundlage dieser Überlegungen formulieren wir als Basishypothese:

#### H 1

Das Ansehen von Hausfrauen in Deutschland wird von der Bevölkerung höher eingeschätzt als das Ansehen von Arbeitslosen, aber geringer als das Ansehen von beruflichen Tätigkeiten.

### Differenzierte Wahrnehmungen des Ansehens von Hausfrauen

Bereits Mitte des letzten Jahrhunderts wurde prominent darauf hingewiesen (z. B. Hyman 1953), dass die Wahrnehmung von sozialem Status auch von der Gruppenzugehörigkeit und Situation der Wahrnehmenden selbst abhängt. Boltanski und Thévenot (1983) beschreiben die differenziellen Bewertungen von Berufsgruppen in diesem Zusammenhang als Machtkämpfe, die in der Gesellschaft ausgetragen werden. Auch empirisch lassen sich unterschiedliche Bewertungsmuster hinsichtlich des Prestiges von Berufen identifizieren, die systematisch an sozialstrukturelle Merkmale der Beurteilenden geknüpft sind. Ein deutlicher Einfluss geht zum einen vom individuellen Bildungsstand aus (Wegener 1992; Zhou 2005; Lynn und Ellerbach 2017), zum anderen vom Geschlecht (Daniel 1979; Valentino 2020). Aus der Forschung zur Arbeitsteilung zwischen Männern und Frauen ist ferner bekannt, dass sowohl die Erwerbsaktivität als auch die Zugehörigkeit zu einer bestimmten Geburtskohorte tendenziell mit unterschiedlichen Präferenzen für ein bestimmtes Modell der Arbeitsteilung einhergehen (vgl. Alwin et al. 1992; Kurz 1998; Ebner et al. 2020). Inwiefern die Beurteilungen des gesellschaftlichen Ansehens von Hausfrauen nach den dargelegten Kriterien ebenfalls systematisch variieren könnten, wird nachfolgend diskutiert.

#### Differenzierungen nach Geburtskohorte

Folgt man dem Paradigma Karl Mannheims (2017), unterscheidet sich das Erleben, Denken, Fühlen und Handeln der Gesellschaftsmitglieder zwischen verschiedenen Generationen zum Teil deutlich. Mit dem Zeitpunkt der Geburt sind ungleiche historische Erfahrungen und erlebte Normalitäten verbunden (vgl. Alwin und McCammon 2003). Häufig wird in diesem Zusammenhang darauf hingewiesen, dass vor allem die Erfahrungen der frühen Lebensjahre individuelle Wertordnungen prägen und sich diese dann über das Leben hinweg nicht mehr sonderlich verändern (vgl. Inglehart 1998; Alwin und McCammon 2003). Obgleich eine Stabilität von Wertorientierungen über den Lebensverlauf durchaus umstritten ist (Davis 2007), sollten sich dennoch substanzielle Unterschiede zwischen den Kohorten zeigen; selbst dann, wenn Kompositionsunterschiede berücksichtigt werden, also die verschiedenartige Zusammensetzung der Geburtsjahrgänge, etwa nach Familienstand oder Bildungsniveau (Firebaugh 1992).

Für ältere Kohorten stellte das Hausfrauenmodell in Kindheit und Jugend eine deskriptive Norm dar, war also weit verbreitete Normalität. Während es Mitte des 20. Jahrhunderts daher nicht zwangsläufig negativ behaftet war, leistet der quantitative Bedeutungsverlust von Hausfrauen nun Marginalisierungstendenzen Vorschub – das goldene Zeitalter der Ehe der 1950er-Jahre ist lange vorüber (Meyer 2014, S. 438 f.). Jüngere Geburtsjahrgänge sind mit der Erwerbstätigkeit von Frauen viel eher vertraut und die Hausfrauentätigkeit wird unter ihnen womöglich als etwas Althergebrachtes angesehen, was sich wiederum negativ auf das zugeschriebene Ansehen auswirken könnte. Aus Untersuchungen geht bereits hervor, dass konservative Sichtweisen auf geschlechterspezifische Arbeit von Jüngeren seltener geteilt werden. Die Berufstätigkeit der Frau findet in der Kohortenabfolge ansteigend Zustimmung (vgl. Baier 2014, S. 261 ff.).

Wir stellen dementsprechend die folgende Hypothese auf:

##### H 2a

Ältere Geburtskohorten schätzen das Ansehen von Hausfrauen in Deutschland höher ein als jüngere Geburtskohorten.

#### Bildungsspezifische Differenzierungen

In kredentialistischen Gesellschaften wie Deutschland sind Bildungssystem und Erwerbssystem eng aneinandergekoppelt (vgl. Allmendinger 1989). Individuen investieren in ihr institutionalisiertes Kulturkapital (Bourdieu 1983), um wettbewerbsfähig zu sein. Nachweislich erhöht hierzulande ein beruflicher Bildungsabschluss die Chancen auf Beschäftigung wie auch auf gute Löhne; besonders günstig sind die Erfolgsaussichten von Akademikern (BA 2019b; Söhnlein et al. 2017, S. 54 f.; Stüber 2017, S. 94 f.). Folglich ist davon auszugehen, dass mit einem beruflichen Abschluss die individuelle Nähe zum Arbeitsmarkt steigt und gleichermaßen die persönliche Distanz zur sozialen Position der Hausfrau, die ihre Arbeit jenseits des Arbeitsmarktes verrichtet. Dies mag zu einer niedrigeren Beurteilung des Hausfrauenansehens führen. Bisherige Studienergebnisse machen außerdem deutlich, dass sich der Bildungsstand von Befragten als ein guter Prädiktor für Geschlechterrolleneinstellungen erweist. In höheren Bildungsgruppen werden traditionelle Vorstellungen zur Arbeitsteilung zwischen Männern und Frauen besonders häufig abgelehnt (vgl. Kurz 1998, S. 179 f.; Diabaté 2015): „That is, level of education matters because through socialization higher educated groups will hold norms and values about gender relations that are substantially different from those of lower educated groups. Whereas for the lower educated inequality between the sexes is given, the higher educated are aiming at gender equality in public and private spheres as much as possible“ (van Berkel und De Graaf 1999, S. 789 f.).

Wir leiten aus diesen Überlegungen die folgende Forschungshypothese ab:

##### H 2b

Je höher das formale Qualifikationsniveau von Personen ist, desto niedriger schätzen sie das Ansehen von Hausfrauen ein.

#### Differenzierung nach Teilhabe am Erwerbssystem

Ein Kernmerkmal von Hausfrauen ist die Nichtteilhabe am Erwerbssystem. Hieran anknüpfend stellt sich die Frage, inwiefern Personen, die selbst erwerbstätig sind, das Ansehen dieser Gruppe anders einschätzen als Personen, die selbst nichterwerbstätig sind. Einen theoretischen Bezugsrahmen stellt die Theorie der sozialen Identität nach Tajfel und Turner (1986) her, die auf der Inspektion von Gruppeninteraktionen und Gruppendistinktion beruht. Demzufolge tendieren Mitglieder der Eigengruppe dazu, sich gegenüber denen der Fremdgruppe abzugrenzen, um ihre als positiv wahrgenommene soziale Identität zu erhalten. „Positive social identity is based to a large extent on favorable comparisons that can be made between the in-group and some relevant out-groups: the in-group must be perceived as positively differentiated or distinct from the relevant out-groups“ (Tajfel und Turner 1986, S. 16). Auch wenn hier durch die Anlage der Befragung eher von unbewussten, verinnerlichten Distinktionsversuchen ausgegangen werden muss, ist eine tendenzielle Abwertung des Ansehens von Nichterwerbstätigen durch Erwerbstätige generell wahrscheinlich. Darüber hinaus zeigen frühere Studien, dass berufstätige Frauen und – mit schwächerer Ausprägung – auch berufstätige Männer weniger konservativ eingestellt sind (vgl. Alwin et al. 1992; Kurz 1998, S. 179) und sich gegenüber weiblicher Erwerbstätigkeit prinzipiell als aufgeschlossener erweisen (Baier 2014, S. 267).

Aus den obigen Ausführungen leiten wir folgende Hypothese ab:

##### H 2c

Erwerbstätige Personen schätzen das Ansehen von Hausfrauen niedriger ein als nichterwerbstätige Personen.

#### Differenzierungen nach Geschlecht und Interaktionseffekte

Sowohl Männer als auch Frauen werden durch die steigende weibliche Erwerbsbeteiligung vor neue Herausforderungen im Haushalts- und Familienkontext gestellt. Das klassische Modell mit männlichem Alleinernährer und weiblicher Familienarbeiterin kann heute im Allgemeinen nur noch wenig soziale Akzeptanz verbuchen (vgl. Diabaté 2015; Beck-Gernsheim 1983). Doch gerade Frauen dürften kritischer mit dem traditionellen Geschlechterrollenleitbild umgehen, welches als Relikt aus der Zeit der *Hausfrauenehe* Mitte des 20. Jahrhunderts angesehen werden kann und gewissermaßen eine „naturrechtliche Begründung“ (Meier-Gräwe 2015, S. 4) für nicht entlohnte Verantwortlichkeiten im Haushalt und in der Familie impliziert (vgl. Brines 1994). Denn die tatsächliche Aufteilung der alltäglichen Zuständigkeiten in der Familie erfolgt häufig zum Nachteil der Frauen (vgl. Dechant et al. 2014). Die mögliche negative Assoziation mit der Hausfrauenrolle, das vermehrte Streben nach Erwerbsarbeit und Karriere sowie nach Emanzipation und (finanzieller) Unabhängigkeit führt, so unsere Annahme, zu einer geringeren Ansehensbewertung von Hausfrauen beim weiblichen Geschlecht. Für Männer dagegen ist Erwerbsarbeit schon lange Normalität und die Arbeit der Frau im Haushalt ist für sie nicht mit den genannten Problemen und Anerkennungsdefiziten behaftet, sondern stellt sogar eine Entlastung dar und könnte daher stärker wertgeschätzt werden.

Wir stellen auf Grundlage dieser Aspekte als Hypothese auf:

##### H 2d

Frauen schätzen das Ansehen von Hausfrauen niedriger ein als Männer.

Da die Kategorie Geschlecht nicht nur für sich genommen erklärungswirksam sein kann, sondern in Anlehnung an den Forschungsstand auch Wechselwirkungen mit anderen sozialstrukturellen Kategorien aufweist (vgl. Nilson 1978), berechnen wir explizit auch Interaktionseffekte. Es ist anzunehmen, dass Männer und Frauen auch in Abhängigkeit ihres Geburtsjahrgangs, ihres jeweiligen Qualifizierungsstands und ihrer Erwerbsaktivität verschiedenartige Wirklichkeitskonstruktionen vornehmen und entsprechend differierende Einschätzungen zum Hausfrauenansehen abgeben.

## Forschungsdesign

### Datensatz und Untersuchungspopulation

Der Datensatz, welcher den statistischen Auswertungen zugrunde liegt, wurde im Rahmen des Projekts „Berufe in Deutschland: Gesellschaftliche Wahrnehmung und Persönlichkeitseigenschaften“ des Bundesinstituts für Berufsbildung (BIBB) erhoben. Hierbei handelt es sich um eine Zusatzerhebung zur jüngsten Erwerbstätigenbefragung (ETB) des BIBB und der Bundesanstalt für Arbeitsschutz und Arbeitsmedizin (BauA),^4^ bei der zwischen Oktober 2017 und Mai 2018 9011 Personen interviewt wurden. Die Interviews wurden vom Sozialwissenschaftlichen Umfragezentrum mittels CATI-Verfahren (Festnetz und Mobilfunk) durchgeführt (vgl. Danullis 2018). Grundgesamtheit der Untersuchung ist die Wohnbevölkerung in Deutschland ab 15 Jahren. Die Stichprobe setzt sich aus zwei, jeweils zufällig gezogenen, Teilstichproben von (wieder-)befragungsbereiten Kernerwerbstätigen und Nichtkernerwerbstätigen^5^ aus der ETB zusammen.^6^ Für die Auswertungen wurde eine Gewichtungsvariable herangezogen, die design- und ausfallbedingte Unterschiede der Stichprobe an die Grundgesamtheit (Referenz: Mikrozensus 2017) anpasst (zur Gewichtung der Daten vgl. Faulbaum 2018).

Die Erhebung beinhaltete unter anderem die Erfassung des Ansehens von unterschiedlichen Berufen in Deutschland sowie von Nichterwerbstätigengruppen. Die hier zentrale Frage zum Ansehen von Hausfrauen wurde einer Zufallsauswahl von 1159 Personen (627 Kernerwerbstätigen und 532 Nichtkernerwerbstätigen) unter den insgesamt 9011 Befragten vorgelegt. Für die Auswertungen im vorliegenden Papier wird die Untersuchungsgruppe auf Fälle mit vollständigen Angaben zu den einbezogenen Variablen beschränkt. Dadurch reduziert sich die Stichprobengröße auf 1089 Fälle, die in der Tab. 2 im Anhang deskriptiv beschrieben werden.

### Variablenauswahl und Operationalisierungen

Die zentrale *abhängige Variable* der Untersuchung ist die Einstufung des gesellschaftlichen Ansehens von Hausfrauen, welche durch folgende Formulierung erfragt wurde: „Denken Sie nun bitte an Hausfrauen, also solche Frauen, die sich vor allem um Haus- und Familienarbeit kümmern und keiner oder nur geringfügiger Lohnarbeit nachgehen. Wie hoch ist Ihrer Meinung nach heute das Ansehen von Hausfrauen in Deutschland? Geben Sie bitte einen Wert von 0 bis 10 an. 0 bedeutet, dass Hausfrauen in Deutschland ein „sehr geringes Ansehen“ haben und 10 ein „sehr hohes Ansehen“. Mit den Werten dazwischen können Sie Ihre Meinung abstufen.“

Mithilfe dieser Variable, die wir als quasi-metrisch betrachten, kann die erste forschungsleitende Frage zum allgemeinen Ansehen von Hausfrauen in Deutschland beantwortet werden. Im Unterschied zu objektiven Messmethoden, bei denen die sozialstrukturelle Einstufung in der Regel durch den Forscher anhand von Indikatoren wie Beruf, Einkommen und Bildung erfolgt und die bei Statusskalen wie dem ISEI häufig eingesetzt werden, wird die gesellschaftshierarchische Einordnung bei Prestigemessungen üblicherweise durch die direkte Abfrage von Einschätzungen ermittelt. Im vorliegenden Fall handelt es sich aber weder um eine Selbstverortung (z. B.: „Welcher Schicht rechnen Sie sich selbst zu?“) noch geht es um die Abfrage der persönlichen Meinung nach eigenen normativen Maßstäben („Wie hoch *sollte* das Ansehen sein?“), sondern es wird die subjektive Methode der Fremdeinschätzung angewandt (vgl. Tegtmeyer 1979, S. 73). Trotz der Gefahr, dass „die Einschätzung anderer … von der eigenen sozialen Stellung mehr oder weniger stark beeinflußt wird“ (Tegtmeyer 1979, S. 74), ist ein großer Vorteil bei dieser Herangehensweise im Ergebnis eine umfassende Prestigebewertung aus aggregierten Einzelwahrnehmungen, „die zwar auf subjektiven Urteilen aufbaut, deren Interpretation und soziologische Bedeutung sich jedoch an strukturell-hierarchischen Gesichtspunkten orientier[t]“ (Wegener 1988, S. 223) und somit eine intersubjektive Realität abbildet. Prestige kann in diesem Sinne als „Zwitterwesen“ (Wegener 1985, S. 209) betrachtet werden, da es zwar eine „subjektiv-symbolische Bedeutung“ hat (Wegener 1985, S. 210), also subjektiv erlebt und zugeschrieben wird, sich aber dennoch an objektiven Statusmerkmalen orientiert und, so vermuten wir, in der Gesellschaft etablierte Wertorientierungen und Leitbilder widerspiegelt. Eine weitere Besonderheit von Prestigemessungen ist, dass Inhaber von sozialen Positionen nicht als Individuen, sondern als „statistisches Kollektiv“ (Tegtmeyer 1979, S. 73) behandelt werden, also einer Gruppe von Personen mit ähnlichem beruflichen oder sozialen Hintergrund angehören.

Die zweite forschungsleitende Frage dieses Beitrags zielt darauf ab, herauszufinden, ob die Wahrnehmung des Hausfrauenansehens auch von der Zugehörigkeit zu einer sozialen Gruppe abhängt. Als zentrale *unabhängige Variablen* werden in diesem Zusammenhang die folgenden kategorialen Variablen betrachtet: Die Variable *Geburtskohorte* teilt die Befragten anhand ihres Geburtsjahres in sieben Kohorten ein: vor 1950 (Referenzkategorie), 1950–1959, 1960–1969, 1970–1979, 1980–1989, 1990–1999, 2000 und später. Die Ursprungsvariable zum *höchsten Ausbildungsabschluss* wurde wie folgt recodiert: kein beruflicher Abschluss (Referenzkategorie), noch kein beruflicher Abschluss (z. B. Schüler), abgeschlossene Berufsausbildung, Meister‑/Techniker‑/Fortbildungsabschluss, Fachhochschul‑/Universitätsabschluss. Hinsichtlich der Variable *Erwerbstätigkeit* wird in den Analysen zwischen nichterwerbstätig (Referenzkategorie) und erwerbstätig unterschieden.^7^ Das *Geschlecht* der Befragten wurde binär erfasst und hat nach Umcodierung der Variable die Merkmalsausprägungen männlich (Referenzkategorie) und weiblich.

Als *Kontrollvariablen* gehen in die statistische Datenanalyse außerdem die folgenden dichotomisierten Variablen ein: Familienstand (nicht verheiratet [Referenzkategorie], verheiratet), Staatsangehörigkeit^8^ (deutsch [Referenzkategorie], nicht deutsch), Region als Wohnort der Befragten in Ost- oder Westdeutschland (neue Bundesländer [Referenzkategorie], alte Bundesländer) sowie im städtischen oder ländlichen Raum^9^ (Stadt [Referenzkategorie], Land).

### Analysestrategie und statistische Methoden

Für die generelle Einordnung des gesellschaftlichen Ansehens von Hausfrauen wird die Verteilung der Ansehenseinschätzungen (Skala 0–10) zunächst univariat ausgewertet, der Durchschnitt und die Standardabweichung berechnet. Das durchschnittliche Ansehen von Hausfrauen wird darüber hinaus mit dem Ansehen von Arbeitslosen sowie von beruflichen Tätigkeiten verglichen, die nach der Klassifikation der Berufe von 2010 formal vier unterschiedlichen Anforderungsniveaus zugeordnet werden können: 1) Helfer- und Anlerntätigkeiten, für die in der Regel kein formaler beruflicher Bildungsabschluss vorausgesetzt wird; 2) fachlich ausgerichtete Tätigkeiten, die üblicherweise einen Berufsabschluss voraussetzen; 3) komplexe Spezialistentätigkeiten, die häufig eine berufliche Fort- oder Weiterbildung erfordern (Meister, Techniker, Fachwirte u. Ä.); 4) hochkomplexe Tätigkeiten, die eine Hochschulausbildung oder entsprechende Berufserfahrung erfordern (BA 2011, S. 27 f.). Mit diesem Vorgehen kann die Basishypothese der Studie (*H 1*) getestet werden.

Im Anschluss werden erste bivariate Zusammenhänge zwischen Einzelvariablen ermittelt sowie entsprechende Signifikanztests durchgeführt. Mittels multivariater linearer Regressionsanalysen untersuchen wir schließlich in Modell 1, ob personenbezogene Merkmale (Kohorte, Ausbildung, Erwerbstätigkeit, Geschlecht) die Beurteilung des Ansehens von Hausfrauen signifikant beeinflussen (Hypothesen *2a* bis *2d*). Die Hypothesentests erfolgen unter Einbeziehung der oben aufgeführten Kontrollvariablen. In weiteren Modellen nehmen wir Interaktionsterme zwischen Geschlecht und Geburtskohorte (Modell 2), Geschlecht und Bildung (Modell 3) sowie Geschlecht und Erwerbsbeteiligung (Modell 4) zusätzlich zu den Haupteffekten mit auf (siehe Tab. 4 im Anhang). Dadurch sollen mögliche weitere geschlechtsspezifische Unterschiede in der Wahrnehmung des Hausfrauenansehens aufgedeckt werden.

Univariate, bivariate und multivariate Analysen erfolgen stets gewichtet. Alle Datenanalysen werden mithilfe der Statistiksoftware Stata, Programmversion 15, durchgeführt.

## Untersuchungsergebnisse

### Befunde der deskriptiven Statistik

Das Ansehen von Hausfrauen wurde von den 1089 Befragten im Durchschnitt mit rund 4,75 Ansehenspunkten (gewichtet) bewertet. Damit liegt das Ansehen etwas unterhalb des Skalenmittelpunkts von 5. Die Standardabweichung beträgt 2,34. Es wurden sämtliche Werte der Skala von 0 bis 10 vergeben, wobei sehr hohe Werte eher selten genannt wurden, häufiger aber Werte am linken Rand, sodass die Verteilungskurve leicht rechtsschief verläuft (Schiefe: 0,27).

**Figure 1:**
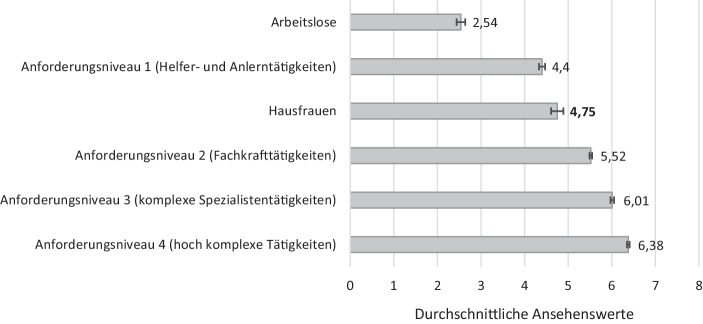

Aufschlussreich ist darüber hinaus der Vergleich zwischen den Ansehenseinschätzungen für Hausfrauen mit denen für Arbeitslose und für Berufe mit unterschiedlichen Anforderungsniveaus (vgl. Abb. 1; Tab. 2 im Anhang). Hier wird deutlich, dass das Hausfrauenansehen durchaus über dem Ansehen von Arbeitslosen (MW = 2,54) liegt, so wie es auch in der ersten Forschungshypothese (*H 1*) formuliert wurde. Der Vergleich mit beruflichen Tätigkeiten verschiedener Anforderungsniveaus ist dagegen differenzierter zu beurteilen. Zwar liegt das durchschnittliche Ansehen von fachlich ausgerichteten Tätigkeiten (MW = 5,52), von komplexen Spezialistentätigkeiten (MW = 6,01) sowie von hochkomplexen Tätigkeiten (MW = 6,38), wie theoretisch angenommen, klar über dem der Hausfrauen. Allerdings wird das Hausfrauenansehen leicht höher beurteilt als das von Helfer- und Anlerntätigkeiten (MW = 4,40). Alle Mittelwertunterschiede erweisen sich durchgängig als höchst signifikant.

Für die Hypothesen *H 2a* bis *H 2d* liefert die bivariate Betrachtung (vgl. Tab. 3 im Anhang) bereits erste Indizien. Bei den Ansehensbewertungen nach Geburtskohorte ist auffällig, dass die beiden Kategorien an den äußeren Rändern (vor 1950 sowie 2000 und später) die höchsten Mittelwerte aufweisen. Der niedrigste Wert ist in der mittleren Kategorie (1970–1979) zu finden, gefolgt von den Vorgängerkohorten der Geburtsjahrgänge 1960–1969 und 1950–1959. Aufsteigend schließen sich hieran die jüngeren Kohorten der 1990–1999 Geborenen und der Jahrgänge 1980–1989 an. Unsere Vermutung, dass ältere Geburtsjahrgänge das Ansehen von Hausfrauen grundsätzlich höher einschätzen als jüngere (*H 2a*), lässt sich somit nicht bestätigen, da gerade bei den Jüngeren wieder eine Zunahme der Werte zu verzeichnen ist. Innerhalb der jüngsten Kohorte ist die Streuung mit einer Standardabweichung von 3,0 allerdings auch am höchsten und das Ergebnis ist aufgrund der geringen Fallzahlen in dieser Gruppe mit Vorsicht zu interpretieren. Insgesamt erweisen sich die Mittelwertunterschiede auf Basis einer Varianzanalyse aber als höchst signifikant.

Gleiches gilt auch für die Einschätzungen des Hausfrauenansehens nach Ausbildungsabschluss. Hier vergeben Befragte, die keinen beruflichen Abschluss oder noch keinen beruflichen Abschluss besitzen, die höchsten Werte. Personen mit abgeschlossener Berufsausbildung und mit Fachhochschul‑/Universitätsabschluss schätzen das Ansehen am niedrigsten ein. In der Tendenz lässt sich feststellen, dass das Ansehen von Hausfrauen mit steigender formeller Bildung der Befragten signifikant niedriger wahrgenommen wird, was auf eine Bestätigung der Forschungshypothese *H 2b* hindeutet. Eine Abweichung hiervon stellt jedoch die Kategorie der Personen mit Meister‑/Techniker‑/Fortbildungsabschluss dar, die das Ansehen vergleichsweise hoch beurteilen (wenn auch, wie angenommen, immer noch niedriger als Personen ohne beruflichen Abschluss). Die Mittelwertunterschiede auf Basis einer Varianzanalyse sind auch in diesem Fall höchst signifikant.

Bei der dichotomisierten Variable Erwerbstätigkeit ergibt sich ein eindeutiges Bild: Nichterwerbstätige schätzen das Hausfrauenansehen höher ein als Erwerbstätige, womit sich eine Bestätigung der aufgestellten Hypothese (*H 2c*) andeutet. Der durchgeführte t‑Test weist auf einen höchst signifikanten Unterschied hin.

Beim Vergleich der Geschlechterkategorien ist ebenfalls ein relativ großer Unterschied zwischen den vergebenen Ansehenswerten zu erkennen: Männer beurteilen das Hausfrauenansehen höher als Frauen. Aufgrund dieser höchst signifikanten Bewertungsdifferenzen wird auch die Hypothese *H 2d* auf Grundlage der bivariaten Statistik bekräftigt.

### Befunde der multivariaten Statistik

Der Vergleich der Mittelwerte sowie die durchgeführten Signifikanztests verdeutlichten bereits, dass zwischen den unabhängigen Variablen und der abhängigen Variable statistische Zusammenhänge bestehen. WeIche Größen auch unter Kontrolle weiterer Variablen ihren Einfluss behalten, wurde im Anschluss mithilfe linearer Regressionsmodelle überprüft.

Die multiple Regression (vgl. Tab. 1) zeigt, dass die Einschätzungen zum Ansehen von Hausfrauen zwischen Personen ohne beruflichen Abschluss, mit Berufsausbildung, Fortbildungsabschluss und akademischem Abschluss, zwischen erwerbstätigen und nichterwerbstätigen Personen sowie zwischen Frauen und Männern, auch unter Einbeziehung der Kontrollvariablen, zum Teil höchst signifikant voneinander abweichen. Bei den Ansehensurteilen nach Geburtskohorte lassen sich jedoch lediglich bei den ersten drei Kategorien signifikante Unterschiede im Vergleich zur Referenzkategorie (Geburt vor 1950) beobachten: Die Befragten der Geburtskohorten ab 1950 bis 1979 nehmen das Ansehen von Hausfrauen zunehmend als geringer wahr. Bei den 1970–1979 Geborenen wird es gar um 1,27 Punkte niedriger eingeschätzt. Bei den Kohorten ab 1980 gibt es schließlich keinen signifikanten Unterschied mehr zur Referenzgruppe. Die ab 2000 Geborenen weisen sogar fast identische Werte wie die vor 1950 Geborenen auf. Diese Ergebnisse deuten also über die Kohorten hinweg auf einen U‑förmigen Verlauf des wahrgenommenen Hausfrauenprestiges hin. Es bleibt zu konstatieren, dass, wie *H 2a* impliziert, das Ansehen von Hausfrauen in Abhängigkeit der Geburtskohorte beziehungsweise des Alters der Befragten unterschiedlich eingeschätzt wird – allerdings nicht im Sinne einer linearen Abnahme im Kohortenverlauf. Im Vergleich zu Befragten, die keinen beruflichen Abschluss besitzen, schätzen Befragte mit einem beruflichen Abschluss das Ansehen von Hausfrauen signifikant niedriger ein. Bei Vorliegen eines Fachhochschul- oder Universitätsabschlusses beträgt der Wert fast einen Ansehenspunkt (0,93) weniger. Dies entspricht der in *H 2b* getroffenen Annahme. Doch auch hier bilden die Ergebnisse, anders als vermutet, keinen ganz klar abnehmenden Verlauf mit höherer Bildung ab. Befragte, die noch keine Berufsausbildung vorweisen können, weil sie z. B. Schüler, Auszubildende oder Studierende sind, schätzen das Ansehen von Hausfrauen sogar um fast einen Ansehenspunkt (0,95) niedriger ein.

**Table 1:** 

Erklärende Variablen und Ausprägungen	Koeff.	Standardfehler
**Geburtskohorte**		
*Vor 1950 (Ref.)*		
1950–1959	−0,87**	0,27
1960–1969	−1,02***	0,29
1970–1979	−1,27***	0,30
1980–1989	−0,19	0,31
1990–1999	−0,63	0,33
2000 und später	0,06	0,54
**Ausbildung**		
Kein beruflicher Abschluss (Ref.)	−	–
Noch kein beruflicher Abschluss	−0,95**	0,34
Abgeschlossene Berufsausbildung	−0,81***	0,22
Meister‑/Techniker‑/Fortbildungsabschluss	−0,77**	0,28
Fachhochschul‑/Universitätsabschluss	–0,93***	0,24
**Erwerbsbeteiligung**		
*Nicht erwerbstätig (Ref.)*		
Erwerbstätig	−0,40*	0,20
**Geschlecht**		
*Männlich (Ref.)*		
Weiblich	−1,04***	0,14
**Familienstand**		
*Nicht verheiratet (Ref.)*		
Verheiratet	−0,03	0,16
**Staatsangehörigkeit**		
*Deutsch (Ref.)*		
Nicht deutsch	0,37	0,31
**Ost/West**		
*Neue Bundesländer (Ref.)*		
Alte Bundesländer	−0,17	0,22
**Städtisch/Ländlich**		
*Stadt (Ref.)*		
Land	0,27	0,15
*N*	1089	
R‑Quadrat	0,12	

Eigene Berechnungen auf Basis des Datensatzes „Ansehen von Berufen in Deutschland 2017/2018“; *n* = 1089

*signifikant (*p* < 0,05), **hoch signifikant (*p* < 0,01), ***höchst signifikant (*p* < 0,001)

Ein weiterer Befund aus der multivariaten Statistik ist, dass Erwerbstätige einen um durchschnittlich 0,40 geringeren Ansehenswert für Hausfrauen vergeben als Nichterwerbstätige. Diese Differenz erweist sich als signifikant, was für die Annahme aus *H 2c* spricht. Schließlich schätzen Frauen das Ansehen von Hausfrauen um 1,04 niedriger ein als Männer. Da dieser Unterschied höchst signifikant ist, lässt sich die in *H 2d* getroffene Vermutung mithilfe der Daten stützen. Die Kontrollvariablen Familienstand (verheiratet/nicht verheiratet) und Staatsangehörigkeit (deutsch/nicht deutsch) sowie die Regionaleinteilungen nach Ost/West und städtisch/ländlich weisen keine statistisch signifikanten Koeffizienten auf. Bei der Stadt/Land-Einteilung ergibt sich jedoch annähernd ein Signifikanzniveau von 5 % (*p* = 0,08), das heißt, in der Tendenz schätzen Befragte aus dem ländlichen Raum das Ansehen von Hausfrauen höher ein (+0,27) als solche aus dem städtischen Raum.

**Figure 2:**
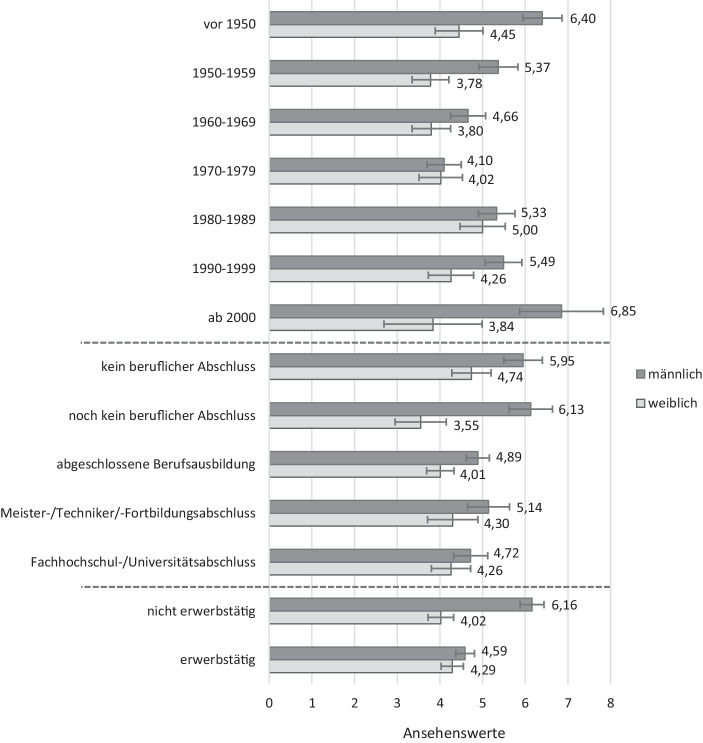

Um Interaktionseffekte zwischen der Variable Geschlecht und den Variablen Geburtskohorte, Ausbildungsabschluss und Erwerbsbeteiligung zu ermitteln, wurden diese nacheinander mit in die Regressionsgleichung einbezogen (vgl. Tab. 4 im Anhang, Modelle 2–4). Zur Erleichterung der Interpretation wurden außerdem jeweils die geschätzten Mittelwerte (vorhergesagte Werte) für die Bewertung des Hausfrauenansehens betrachtet (Abb. 2).

Bei der Kombination aus Geschlecht und Geburtskohorte der Befragten zeigt sich, dass die Unterschiede zwischen Männern und Frauen an den Rändern, bei den vor 1960 Geborenen und dann wieder bei den ab 1990 Geborenen, besonders groß sind. Männer schätzen das Ansehen von Hausfrauen hier deutlich höher ein als Frauen. Zwei weitere Auffälligkeiten sind hervorzuheben: Erstens unterscheiden sich Männer und Frauen der Geburtskohorten 1970–1979 und 1980–1989 in ihren Einschätzungen zum Ansehen kaum und zweitens sind die Beurteilungen der Frauen über alle untersuchten Kohorten hinweg geringeren Schwankungen unterworfen als die der Männer.

Bei den Interaktionseffekten zwischen Geschlecht und Ausbildungsabschluss der Befragten ist die größte Differenz zwischen Frauen und Männern, die noch keinen beruflichen Abschluss besitzen, zu beobachten. In dieser Gruppe beurteilen Frauen das Ansehen von Hausfrauen um 2,58 Punkte niedriger als Männer. Ein signifikanter Unterschied in den Einschätzungen nach Geschlecht ist außerdem bei den Befragten, die keinen beruflichen Abschluss aufweisen, und denen mit Berufsausbildung zu konstatieren – in beiden Fällen schätzen Männer das Hausfrauenansehen höher ein. Kein signifikanter Unterschied zwischen Männern und Frauen zeigt sich hingegen bei den höheren Abschlüssen, das heißt bei Befragten mit Meister‑, Techniker- oder Fortbildungsabschluss sowie bei Befragten mit Fachhochschul- oder Universitätsabschluss. In der letzten Gruppe ist auch die absolute Differenz der Werte mit 0,46 Einheiten am geringsten.

Im letzten Analyseschritt werden die Urteile zum Ansehen von Hausfrauen als Interaktionsergebnis aus Geschlecht und Erwerbsbeteiligung betrachtet. Ein signifikanter Unterschied in der Einschätzung des Ansehens von Hausfrauen zwischen Männern und Frauen lässt sich nur in der Gruppe der nichterwerbstätigen Personen erkennen (2,14). Unter den Erwerbstätigen ist das Bewertungsverhältnis zwischen den Geschlechtern dagegen nahezu ausgeglichen (0,30), die abgegebenen Werte unterscheiden sich hier nicht signifikant voneinander.

## Fazit und Ausblick

Hausfrauen stellen im Zuge der voranschreitenden Teilhabe von Frauen am Erwerbsleben, nicht nur in Deutschland, aktuell zwar eine klare Minderheit unter der weiblichen Bevölkerung dar. Unklar ist aber noch, welcher Status dieser sozialen Gruppe in der Gesellschaft zugeschrieben wird, konkret, welches Ansehen Hausfrauen in Deutschland genießen. Die vorliegende Studie schließt diese Forschungslücke mittels neuer Befragungsdaten und zeigt nicht nur, wie die deutsche Bevölkerung das gesellschaftliche Ansehen von Hausfrauen beurteilt, sondern auch, dass die Wahrnehmungen hierzu in Abhängigkeit individueller Gruppenzugehörigkeiten erheblich variieren können.

Als erstes zentrales Ergebnis der Studie kann festgehalten werden, dass Hausfrauen ein signifikant höheres Ansehen zugeschrieben wird als Arbeitslosen. Hieraus lässt sich schließen, dass reine Haus- und Familienarbeit in der Bevölkerung durchaus anerkannt sind; gleichzeitig nimmt Erwerbsarbeit einen deutlich höheren Stellenwert ein, wenn es sich um qualifizierte berufliche Tätigkeiten handelt, die typischerweise einen Berufsabschluss, einen Meister‑/Techniker‑/Fortbildungsabschluss oder einen Hochschulabschuss erfordern; Helfer- und Anlerntätigkeiten sind dagegen niedriger angesehen als Hausfrauentätigkeiten.

Ein zweites zentrales Ergebnis ist, dass kein universelles Bild der Statusposition Hausfrau in Deutschland vorherrscht, sondern dass verschiedene sozialstrukturelle Merkmale der Befragten zu signifikanten Unterschieden in den Einschätzungen führen. Hinsichtlich der Kategorie *Geburtsjahrgang* konnte auf Basis der bivariaten sowie der multivariaten Analyse ein annähernd U‑förmiges Muster aufgedeckt werden. Während die vor 1950 Geborenen den Hausfrauen noch ein relativ hohes Ansehen zuschreiben, nehmen die Werte mit steigenden Geburtsjahrgängen allmählich ab und liegen in der mittleren Kohorte (1970–1979) auf dem Tiefstand. Fortan nehmen die Werte aber wieder zu. Es kommt also insgesamt zu einer tendenziellen Wiederaufwertung des Hausfrauenprestiges. Hierbei ist kritisch anzumerken, dass die Perspektive auf Kohorten zwar in der Literatur zum Wandel von Einstellungen und Wertorientierungen dominant und auch theoretisch höchst plausibel ist (vgl. Firebaugh 1992; Inglehart 1998), eine empirische Trennung von Kohorten- und Alterseffekten aber im Rahmen des hier vorliegenden Querschnittsdesigns nicht möglich ist. Ebenfalls unterscheidet sich die Beurteilung des gesellschaftlichen Ansehens von Hausfrauen deutlich nach *Ausbildungsstand*. Hier zeigt sich im Ergebnis, dass es weniger auf das Qualifikationsniveau ankommt, sondern vielmehr darauf, ob überhaupt ein beruflicher Ausbildungsabschluss vorliegt oder nicht. Dies stützt unserer Ansicht nach abermals das Bild einer zertifikatsorientierten Erwerbsgesellschaft. Die *Erwerbsbeteiligung* der Befragten ist eine weitere Kategorie, welche die Wahrnehmung des Ansehens von Hausfrauen beeinflusst. So vergeben Erwerbstätige einen signifikant niedrigeren Wert als Nichterwerbstätige. Dahinter könnte sich der Abgrenzungsmechanismus zwischen Eigen- und Fremdgruppe verbergen (vgl. Abschn. 2.3.3). Zu überlegen wäre an dieser Stelle auch, ob sich die Höherbewertung der Eigengruppe als mehr oder weniger bewusster Versuch einer „persönlichen Günstigstellung“ (Wegener 1988, S. 198 f.) deuten lässt, um so einen gefühlten Statusgewinn erzielen zu können (vgl. Tegtmeyer 1979). In Bezug auf das *Geschlecht* der Befragten lässt sich feststellen, dass Frauen das Ansehen von Hausfrauen im Durchschnitt wesentlich schlechter beurteilen als Männer. Womöglich fühlen sich Frauen im Allgemeinen in der Ausübung von Haus- und Familienarbeit von der Gesellschaft eher wenig wertgeschätzt. Die gestiegene Frauenerwerbsbeteiligung bestätigt zudem ihre zunehmende Orientierung am regulären Arbeitsmarkt, wo mit der Berufstätigkeit ein eigenständig erworbenes Sozialprestige einhergeht, was Emanzipation und gewollte Abwendung von der Hausfrauenrolle impliziert.

Die empirischen Befunde machen, drittens, deutlich, dass sich Frauen und Männer in ihren Urteilen zum Ansehen von Hausfrauen nicht nur in der Gesamtbetrachtung, sondern spezifisch auch nach weiteren Gruppenmerkmalen unterscheiden können. So zeigt die geschlechterdifferenzierte Kohortenanalyse den oben beschriebenen U‑förmigen Verlauf klar nur bei den Männern. Frauen schätzen das Ansehen von Hausfrauen sowohl in den älteren als auch in den jüngeren Kohorten als eher gering ein und unterscheiden sich diesbezüglich signifikant von den Männern. Die Frage, ob die Höherbewertung unter jungen Männern einen Wunsch nach traditioneller Arbeitsteilung widerspiegelt, lässt sich auf Grundlage der vorhandenen Datenbasis noch nicht zufriedenstellend beantworten. Die Aufwertung der Hausfrauentätigkeit könnte auch symbolisch zum Ausdruck bringen, dass weibliche Bewerberinnen gerade für jüngere männliche Kohorten zunehmend eine Konkurrenz auf dem Arbeitsmarkt darstellen. Denn noch immer herrscht die gesellschaftliche Erwartung vor, dass der Mann ökonomisch unabhängig und in der Familie zumindest Haupt-, wenn schon nicht Alleinverdiener sein soll (vgl. Lück 2015, S. 242 f.; West und Zimmerman 1987; Berk 1985). Unklar ist ferner, inwiefern es sich möglicherweise um einen geschlechtsspezifischen Alterseffekt handelt. Weitere augenfällige Differenzen in der Beurteilung des Hausfrauenansehens nach Geschlecht zeigen sich in Abhängigkeit des Ausbildungsstands der Befragten. Während bei hoch qualifizierten Männern und Frauen kein signifikanter Unterscheid beobachtet werden kann, wird ein solcher vor allem bei Personen, die keinen oder noch keinen beruflichen Abschluss erworben haben, deutlich sichtbar. Gering qualifizierte Männer schreiben Hausfrauen ein relativ hohes Ansehen zu. Auch Frauen, die kein berufliches Zertifikat erworben haben, vergeben einen eher hohen Ansehenswert (wenn auch signifikant niedriger als Männer), was mit der Nähe zum Hausfrauenstatus zusammenhängen könnte, welcher für diese Gruppe am ehesten eine Alternative zur Ungelerntentätigkeit darstellt. Dagegen vergeben Frauen, die noch keinen beruflichen Abschluss besitzen (aber einen solchen anstreben) eher niedrige Ansehenswerte. Das Streben nach einem Ausbildungsabschluss kann hier als Indiz für eine stärkere Arbeitsmarktorientierung gedeutet werden. Schließlich unterscheiden sich die Urteile von Männern und Frauen auch danach, ob sie selbst erwerbstätig oder nicht erwerbstätig sind. Während die Bewertungen des Ansehens von Hausfrauen zwischen Erwerbstätigen beider Geschlechter nicht signifikant voneinander abweichen, ist der Unterschied zwischen nichterwerbstätigen Frauen und Männern deutlich. In dieser Gruppe vergeben männliche Befragte hohe Ansehenswerte, wohingegen Frauen das Ansehen von Hausfrauen vergleichsweise niedrig einschätzen. Darüber, warum vor allem nichterwerbstätige Männer und Männer ohne berufliche Qualifikation das Ansehen von Hausfrauen relativ hoch beurteilen, kann an dieser Stelle nur spekuliert werden. Beide Gruppen zeichnen sich tendenziell durch eine geringere Arbeitsmarktnähe aus. Während die Erwerbstätigkeit von Frauen aus ihrer Perspektive durchaus als Angriff auf die Norm männlicher Erwerbsarbeit gewertet werden könnte, stellen Hausfrauen in dieser Hinsicht keine Bedrohung dar. Im Sinne des Doing-Gender-Ansatzes (West und Zimmerman 1987) wäre die Höherbewertung des Hausfrauenstatus ein Versuch, die Männerrolle zu stärken oder die nichterfüllten Erwartungen an das männliche Geschlecht zu kompensieren. Auch wenn sich heute ein neues Männer- beziehungsweise Väterleitbild andeutet, welches mit der gesellschaftlichen Erwartung und auch dem persönlichen Wunsch nach mehr Zeit für Kindererziehung einhergeht (vgl. Lück 2015, S. 243), ist das konservative Bild mit Befürchtungen eines Prestigeverlusts bei Reduzierung der Erwerbs- und Erhöhung der Reproduktionsarbeit verbunden.

Jenseits der zentralen unabhängigen Variablen lohnt auch ein Blick auf die bei uns eingesetzten Kontrollvariablen. In der Tendenz zeigt sich eine leicht höhere Einstufung des Hausfrauenansehens in der ländlichen Wohnbevölkerung als in städtischen Umgebungen, sodass über stärker tradierte Einstellungen auf dem Land gemutmaßt werden kann; jedoch verfehlen die Ergebnisse (knapp) die statistische Signifikanz. Entgegen unserer Erwartung waren zwischen den Einschätzungen der Befragten in den alten und neuen Bundesländern ebenfalls keine signifikanten Unterschiede zu erkennen. Eine mögliche Erklärung könnte darin liegen, dass in der Studie nicht der Geburtsort der Befragten erfasst wurde, sondern lediglich der Wohnort zum Zeitpunkt der Erhebung. Gerade die besondere Situation im geteilten Deutschland der Nachkriegszeit mit unterschiedlichen Sozialisationskontexten in Ost und West und auch deutlich höheren Frauenerwerbstätigenquoten im Osten (vgl. Gysi und Meyer 1993; Meyer und Schulze 1993) bietet jedoch Anlass für eine weiterführende Betrachtung.

Mit den Befunden der vorliegenden Studie zum Ansehen von Hausfrauen gehen Implikationen für Gesellschaft und Arbeit einher. Das insgesamt doch eher geringe Ansehen von Hausfrauen in Deutschland, vor allem aus Sicht der Frauen selber, macht die Übernahme einer Hausfrauenrolle auch zukünftig für diese nur wenig attraktiv. Ergebnisse aus aktuellen Studien deuten ferner darauf hin, dass mehrheitlich von Frauen ausgeübte berufliche Tätigkeiten auf Fachkraftniveau, wie etwa im Bereich der Pflege, ein hohes gesellschaftliches Ansehen versprechen (Hall et al. 2021). Da Pflege- und Erziehungsberufe hinsichtlich ihres Tätigkeitsspektrums große Überschneidungen mit klassischen Hausfrauentätigkeiten aufweisen, legt dieser Befund nahe, dass insbesondere Frauen mit beruflichem Abschluss auch in Zukunft eher die Berufs- als die Hausfrauenrolle wählen.

Gleichwohl hält auch diese Alternative für Frauen mehrere Herausforderungen bereit. Es herrscht nach wie vor das normative Leitbild der „Guten Mutter“ vor, die sich stets für ihr Kind aufopfert und ihre eigenen Bedürfnisse hintenanstellt, und der „Rabenmutter“, welche sich zu stark auf ihr Berufsleben konzentriert, obwohl die Erwerbstätigkeit von Müttern in der Öffentlichkeit positiv konnotiert ist (Diabaté 2015, S. 212 ff.). Typische Frauenberufe, welche auch aufgrund noch existenter konventioneller Geschlechterrollenleitbilder gewählt werden (vgl. Dölling 1993), erfüllen zwar einen hohen gesellschaftlichen Nutzen, sind aber vergleichsweise schlecht bezahlt (Kleinjans et al. 2017). Schließlich übernehmen Frauen trotz Erwerbstätigkeit oft einen Großteil der Haus- und Familienarbeit, was zu einer Doppelbelastung aus Erwerbs- und Reproduktionsarbeit führt (Becker-Schmidt 2008). Nach der Arbeit wartet die „zweite Schicht“ zu Hause (Hochschild und Machung 2012). Oft ergibt sich vor allem für Mütter daher auch eine erzwungene Reduktion entlohnter Tätigkeiten und eine geringere Chance, auf höheren Stellungen in Unternehmen vertreten zu sein (vgl. Kreckel 1992, S. 248 f.). Zur Gleichstellung der Geschlechter kann die zukünftige Familienpolitik weiter die Richtung vorgeben und dafür werben, dass neue Programme wie das Elterngeld-Plus und der Partnerschaftsbonus in Anspruch genommen werden. Ein wichtiges Instrument stellt auch nach wie vor der Ausbau von Kinderbetreuungseinrichtungen dar.

Für die zukünftige Forschung ergeben sich aus unseren Ergebnissen weitere Anregungen. So ist der Vergleich des Hausfrauenansehens bis heute weder im internationalen Vergleich noch über die Zeit systematisch untersucht worden. Es stellen sich etwa die Fragen, ob das Ansehen von Hausfrauen in Ländern geringer ausfällt, in denen mehr Frauen auf dem Arbeitsmarkt (in Vollzeit) tätig sind, inwiefern in geschlechteregalitären Wohlfahrtsregimes andere Einschätzungen zum Status der Hausfrau getroffen werden als in eher konservativen Ländern oder ob gemeinhin Unterschiede zwischen verschiedenen Kulturen ermittelt werden können. Auch wenn generell von einer hohen Stabilität von Prestigebewertungen über die Zeit ausgegangen werden kann (vgl. Hodge et al. 1964; Blau und Duncan 1967; Nakao und Treas 1994), so ist doch mit länger- oder kurzfristigen Veränderungen der Sichtweisen auf einzelne Berufe (vgl. Tegtmeyer 1979, S. 75 f.) und auch auf Tätigkeiten außerhalb des klassischen Erwerbssystems, wie die Reproduktionsarbeit, zu rechnen – beispielsweise im Zusammenhang mit der Covid-19-Pandemie. Da Prestigeurteile „in unserer komplexen Gesellschaft eine Orientierungsfunktion für den Einzelnen“ ausüben (Tegtmeyer 1979, S. 72), sowohl zu objektiver als auch subjektiver Ungleichheit führen und Berufsentscheidungen beeinflussen können, ist es wichtig, Prestigemessungen in regelmäßigen Abständen durchzuführen. Nicht nur Analysen zu unterschiedlichen Geburtskohorten, sondern auch zu zeitlichen Trends (Periodeneffekte) wären für die Ungleichheitsforschung ein klarer Gewinn.

Reproduktionsarbeit jenseits des Marktes wird nach wie vor in ungenügender Weise als bedeutsame gesellschaftliche Leistung anerkannt (vgl. Meier-Gräwe 2015). So ist zum jetzigen Zeitpunkt auch die systematische Erfassung des Prestiges von Nichterwerbstätigengruppen, anders als die Untersuchung von Berufsprestige (vgl. Ebner und Rohrbach-Schmidt, im Erscheinen), quasi nichtexistent. Ein erster Ansatzpunkt wäre daher die Erweiterung der wissenschaftlichen Klassifizierung von Berufen (und damit ausschließlich Erwerbstätigkeit) um Care-Arbeit und ehrenamtliches Engagement. Auf dieser Basis könnten auch die bereits existierenden, auf Erwerbstätigkeit fußenden, Ungleichheitskonzepte und -maße (Klassen, sozioökonomische Indizes) sinnvoll erweitert werden.

Geschlechter- und Familienleitbilder werden zwar in der und durch die Gesellschaft (re-)produziert, können aber kontextspezifisch vor dem Hintergrund unterschiedlicher kultureller Wertmuster und sozialer Milieus (z. B. Akademiker) variieren (vgl. Lück und Diabaté 2015, S. 23 f.; Schiefer und Naderi 2015, S. 158). Zudem unterscheiden sich persönliche und allgemeine Leitbilder zum Teil deutlich. Dadurch kann sowohl für Frauen als auch für Männer ein öffentlicher Druck entstehen, sich an geltende normative Vorstellungen anpassen zu müssen, obwohl das persönlich als ideal empfundene Lebensmodell hiervon abweicht und häufig schon „moderner“ oder „liberaler“ ist (Diabaté et al. 2015, S. 278). Etablierte Denk- und Verhaltensmuster werden schließlich als „Mehrheitsmeinung“ zu einer eigenen Realität (Lück und Diabaté 2015, S. 25). Die Frage, welchen Anteil Geschlechterrollenbilder und -identitäten an Prestigewahrnehmungen haben, ließe sich auf Basis qualitativ angelegter Forschungsdesigns noch detaillierter nachzeichnen. All dies könnte zu einem besseren Verständnis der symbolischen Dimension sozialer Ungleichheit beitragen.

## Anhang

**Table 2:**

Merkmal	Absolute Zahl	Mittelwert (Std. Abw.)/% gerundet
Ansehen Hausfrau	1089	4,75 (2,34)
Ansehen Arbeitslose	1089	2,54 (1,68)
Ansehen berufl. Tätigkeiten AN1	3721^a^	4,40 (2,14)
Ansehen berufl. Tätigkeiten AN2	18.661	5,52 (2,06)
Ansehen berufl. Tätigkeiten AN3	9565	6,01 (2,06)
Ansehen berufl. Tätigkeiten AN4	12.886	6,38 (2,05)
Befragte zum Ansehen der Hausfrau	1089	100
*Geburtskohorte*		
Vor 1950	93	8,5
1950–1959	251	23,0
1960–1969	266	24,4
1970–1979	150	13,8
1980–1989	156	14,3
1990–1999	149	13,7
2000 und später	24	2,2
*Ausbildung*		
Kein beruflicher Abschluss	64	5,9
Noch kein beruflicher Abschluss	89	8,2
Abgeschlossene Berufsausbildung	407	37,4
Meister‑/Techniker‑/Fortbildungsabschluss	123	11,3
Fachhochschul‑/Universitätsabschluss	406	37,3
*Erwerbsbeteiligung*		
Nicht erwerbstätig	468	43,0
Erwerbstätig	621	57,0
*Geschlecht*		
Männlich	598	54,9
Weiblich	491	45,1
*Familienstand*		
Nicht verheiratet	603	55,4
Verheiratet	486	44,6
*Staatsangehörigkeit*		
Deutsch	1048	96,2
Nicht deutsch	41	3,8
*Wohnort Ost/West*		
Neue Bundesländer	146	13,4
Alte Bundesländer	943	86,6
*Wohnort städtisch/ländlich*		
Stadt	775	71,2
Land	314	28,8

^a^Absolute Zahl bezieht sich bei Ansehen berufliche Tätigkeiten AN1–AN4 auf die Anzahl der Ratings

**Table 3:**

	MW	Std. Abw.	Sig.
Gesamt	4,75	2,34	–
*Geburtskohorte*			
Vor 1950	5,60	2,26	F = 10,06***
1950–1959	4,53	2,14	
1960–1969	4,27	2,13	
1970–1979	4,07	2,21	
1980–1989	5,20	2,68	
1990–1999	5,01	2,13	
2000 und später	5,58	3,00	
*Ausbildung*			
Kein beruflicher Abschluss	5,35	2,58	F = 5,19***
Noch kein beruflicher Abschluss	5,06	2,46	
Abgeschlossene Berufsausbildung	4,52	2,45	
Meister‑/Techniker‑/Fortbildungsabschl.	4,80	1,94	
Fachhochschul‑/Universitätsabschluss	4,53	1,90	
*Erwerbsbeteiligung*			
Nicht erwerbstätig	5,18	2,45	t = 4,21***
Erwerbstätig	4,46	2,22	
*Geschlecht*			
Männlich	5,18	2,26	t = 7,87***
Weiblich	4,19	2,33	

Eigene Berechnungen auf Basis des Datensatzes „Ansehen von Berufen in Deutschland 2017/2018“; *n* = 1089

*signifikant (*p* < 0,05), **hoch signifikant (*p* < 0,01), ***höchst signifikant (*p* < 0,001)

**Table 4:**

Erklärende Variablen und Ausprägungen	Koeff.			
	1. Modell	2. Modell	3. Modell	4. Modell
**Geburtskohorten**				
*Vor 1950 (Ref.)*				
1950–1959	−0,87**	−0,97**	−0,77**	−0,90***
1960–1969	−1,02***	−1,48***	−0,92**	−1,08***
1970–1979	−1,27***	−2,03***	−1,19***	−1,34***
1980–1989	−0,19	−0,88*	−0,10	−0,21
1990–1999	−0,63	−0,97*	−0,71*	−0,74*
2000 und später	0,06	0,49	0,05	0,03
**Ausbildung**				
*Kein beruflicher Abschluss (Ref.)*				
Noch kein beruflicher Abschluss	−0,95**	−0,98**	−0,88**	−0,34
Abgeschlossene Berufsausbildung	−0,81***	−0,82***	−0,65**	−0,98***
Meister‑/Techniker‑/Fortbildungsabschluss	−0,77**	−0,83**	−0,70*	−0,95**
Fachhochschul‑/Universitätsabschluss	−0,93***	−0,98***	−0,81***	−1,29***
**Erwerbsstatus**				
*Nicht erwerbstätig (Ref.)*				
Erwerbstätig	−0,40*	−0,40*	−1,27***	−0,35
**Geschlecht**				
*Männlich (Ref.)*				
Weiblich	−1,04***	−1,96***	−2,14***	−1,204
**Familienstand**				
*Nicht verheiratet (Ref.)*				
Verheiratet	−0,03	−0,08	−0,12	−0,02
**Staatsangehörigkeit**				
*Deutsch (Ref.)*				
Nicht deutsch	0,37	0,41	0,42	0,49
**Wohnort Ost/West**				
*Neue Bundesländer (Ref.)*				
Alte Bundesländer	−0,17	−0,17	−0,12	−0,15
**Wohnort städtisch/ländlich**				
*Stadt (Ref.)*				
Land	0,27	0,26	0,29	0,25
**Geschlecht#Kohorte**				
*Weiblich#vor 1950 (Ref.)*				
Weiblich#1950–1959	–	0,38	–	–
Weiblich#1960–1969	–	1,10*	–	–
Weiblich#1970–1979	–	1,88***	–	–
Weiblich#1980–1989	–	1,62**	–	–
Weiblich#1990–1999	–	0,74	–	–
Weiblich#2000 und später	–	−1,05	–	–
**Geschlecht#Bildung**				
*Weibl.#kein beruflicher Abschluss (Ref.)*				
Weibl.#noch kein beruflicher Abschluss	–	–	−1,37**	–
Weibl.#abgeschlossene Berufsausbildung	–	–	0,33	–
Weibl.#Meister‑/Techniker‑/Fortbildungsabschluss	–	–	0,37	–
Weibl.#Fachhochschul‑/Universitätsabschluss	–	–	0,75	–
**Geschlecht#Erwerbstätigkeit**				
*Weiblich#nichterwerbstätig (Ref.)*				
Weiblich#erwerbstätig	–	–	–	1,84***
*N*	1089	1089	1089	1089
R‑Quadrat	0,12	0,15	0,14	0,16

Eigene Berechnungen auf Basis des Datensatzes „Ansehen von Berufen in Deutschland 2017/2018“; *n* = 1089

*signifikant (*p* < 0,05), **hoch signifikant (*p* < 0,01), ***höchst signifikant (*p* < 0,001)

## Footnotes

1 Im Bezugszeitraum 1995 bis 2015 ist zwar weltweit, analog zur Gesamterwerbstätigenquote, ein leichter Rückgang der Frauenerwerbstätigenquote (von rund 52 auf rund 50 %) zu konstatieren. Vor allem in Nord‑, Süd- und Westeuropa nahm die Zahl der Frauen auf dem Arbeitsmarkt jedoch kontinuierlich zu, während die der Männer zeitweise leicht abnahm. Durch diese Entwicklungen hat sich die *Gender Employment Gap* insgesamt verringert (ILO 2016, S. 6 f.).

2 Unter anderem Berk (1985), Blood und Wolfe (1960), Becker (1985), Goffman (1977), van Berkel und De Graaf (1999), Dechant et al. (2014), Evertsson und Nermo (2007), Brines (1994), Gershuny et al. (2005), Carriero und Tedesco (2018), Altintas und Sullivan (2016), Baxter et al. (2015), Alwin et al. (1992), Kurz (1998), Haberkern (2007), Ebner et al. (2020) und Mandel et al. (2020).

3 Neben dem gesetzlichen Mutterschutz für berufstätige Frauen in Deutschland (MuSchG) werden seit 1989 Kinderbetreuungszeiten bei der Rente berücksichtigt (vgl. § 56, § 249 SGB VI). Im Jahr 2014 wurde das Gesetz zur Erhöhung der Mütterrente erweitert (§ 307 d SGB VI; Gesetze: Bundesministerium der Justiz und für Verbraucherschutz/Bundesamt für Justiz 2021). Zudem existieren verschiedene finanzielle Förderinstrumente wie Elternzeit oder Elterngeld. Das Elterngeld-Plus und der Partnerschaftsbonus begünstigen eine partnerschaftlich geteilte Erwerbs- oder Familienarbeit und einen möglichst schnellen Wiedereinstieg in den Arbeitsmarkt (vgl. BMFSFJ 2019; Juncke et al. 2018, S. 16 ff.). Männer gehen jedoch noch immer deutlich seltener in Elternzeit als Frauen, auch wenn ihre Zahl tendenziell zunimmt (Juncke et al. 2018, S. 16).

4 Die Erwerbstätigenbefragungen werden seit 1979 im Abstand von sechs bis sieben Jahren vom BIBB und dem Institut für Arbeitsmarkt und Berufsforschung und seit 2006 mit der BAuA durchgeführt. Die Stichprobe umfasste zuletzt rund 20.000 Erwerbstätige in Deutschland (vgl. Rohrbach-Schmidt und Hall 2020).

5 Bei den Kernerwerbstätigen handelt es sich um deutschsprachige Erwerbstätige (ohne Auszubildende), die zum Zeitpunkt der Befragung eine bezahlte Tätigkeit mit einer Arbeitszeit von mindestens 10 Stunden pro Woche ausübten. Bei den Nichtkernerwerbstätigen handelt es sich um Personen, die in keinem Beschäftigungsverhältnis standen oder einer bezahlten Tätigkeit mit einer durchschnittlichen Arbeitszeit von unter 10 Stunden pro Woche nachgingen (Danullis 2018).

6 Die Auswahl der zu kontaktierenden Telefonnummern für das ETB-Interview wurde mittels Random-Digit-Dialing-Verfahren nach ADM-Standard durchgeführt (Gensicke und Tschersich 2018). Die Ausschöpfungsquote der ETB liegt bei rund 11 bzw. 43 % (Gensicke und Tschersich 2018), die der Zusatzbefragung bei rund 53 bzw. 83 % (Danullis 2018).

7 Nichterwerbstätigkeit schließt geringfügige Beschäftigung unter 10 Stunden pro Woche ein. Ferner kann bei den Nichterwerbstätigen als Status theoretisch differenziert werden: Schüler/Auszubildende/Studierende, Ruheständler/Rentner, Arbeitslose/Arbeitssuchende, Hausfrauen/-männer. Aufgrund geringer Fallzahlen in den Einzelkategorien haben wir uns dazu entschlossen, diese Gruppen zusammen zu betrachten.

8 Die Staatsangehörigkeit liegt als Variable für alle Befragten vor. Die in anderen Studien verbreitete Kategorisierung nach Migrationshintergrund kann nur für die Gruppe der erwerbstätigen Befragten angegeben werden, sodass hier die Staatsangehörigkeit verwendet wird.

9 Die Einteilung erfolgte letztendlich anhand von Gemeindekennziffern (Gebietsstand 2015) mit folgendem Schlüssel: Stadt = 20.000 bis 500.000 und mehr Einwohner, Land = unter 2000 bis unter 20.000 Einwohner. Zuvor wurde eine differenziertere Analyse nach Einwohnerzahl mit fünf Variablenausprägungen vorgenommen. Da sich hier jedoch keine nennenswerten Unterschiede zeigten, fiel die Wahl auf die zwar gröbere, inhaltlich aber zweckmäßige Stadt/Land-Einteilung (in ländlichen Regionen lassen sich mutmaßlich konservativere Beurteilungsmuster aufdecken als in städtischen).
